# Use of virtual reality medical hypnosis for anxiolytic purposes during frozen embryo transfer: A prospective pilot study

**DOI:** 10.1371/journal.pone.0350101

**Published:** 2026-05-26

**Authors:** Octavia Pingault, Annaëlle Testud, Elsa Labrune, Bruno Salle, Mehdi Benchaib, Éloïse Fraison

**Affiliations:** 1 Department of Gynecology-Obstetric and Reproductive Medicine, University Hospital of Lyon, Lyon, France; 2 Research on Healthcare Performance (RESHAPE, Inserm U1290), Université Claude Bernard, Lyon, France; 3 Claude Bernard University, Faculty of Medicine Laennec, Lyon, France; 4 INSERM Unit 1208, Bron, France; 5 Claude Bernard University, Faculty of Medicine Lyon Sud, Lyon, France; 6 UMR, CNRS 5558, LBBE, Villeurbanne, France; Faculty of Medicine Universitas Indonesia, INDONESIA

## Abstract

**Objective:**

To assess the feasibility of using a virtual reality medical hypnosis (VRH) device for anxiolytic purposes during frozen embryo transfer (FET) and to observe its effect on subsequent pregnancy rate.

**Design:**

This is a prospective, single-center study conducted in a French public reproductive medicine ward between December 2023 and 1 May 2024.

**Subjects:**

Women aged 18–45 years who undergoing IVF at the center. The present study included 50 women who had a scheduled FET.

**Interventions:**

Study participants were using the VRH device during the FET.

**Main outcomes measures:**

Feasibility was defined as optimal and incident-free device use. Anxiety levels were measured pre- and post-procedure using the State-Trait Anxiety Inventory (STAI-Y1). Women and caregiver satisfaction were assessed using Likert scales, while acceptability of the procedure was assessed by recording adverse events. HCG test was conducted afterward and compared with a concurrent non-exposed cohort from the same unit and inclusion period using causal inference to analyze pregnancy rates.

**Results:**

50 women were included from the active patient file undergoing FET at the center. The feasibility of using the VRH device was assessed at 96%, with 100% acceptability of the procedure. Women satisfaction was 98%, and caregiver satisfaction reached 100%, with a favorable device’s use opinion in routine practice. A significant reduction in anxiety was observed, with a mean anxiety score of 28.8/80 after the procedure compared to 39.3/80 before (p < 0.001). Regarding pregnancy outcomes, 25 women had a positive HCG test after the procedure, including 20 with HCG > 100 IU/L, resulting in a pregnancy rate of 40%. Although a positive trend was observed in the comparison with the concurrent non-exposed cohort, the difference was not statistically significant (OR 1.18, 95% CI 0.65–2.14; p = 0.583).

**Conclusion:**

The use of a VRH device during FET is feasible and safe, suggesting a promising anxiolytic effect and a useful non-medical tool for optimizing care pathways in assisted reproduction. Further larger and controlled studies are warranted to confirm these encouraging findings and to more thoroughly assess the impact on pregnancy outcomes.

## Introduction

According to the World Health Organization (WHO) [1], infertility rate is around 17.5% worldwide. In France, around 1 in 4 couples is affected. The use of Assisted Reproductive Techniques (ART) have become an unavoidable response for these couples and is constantly increasing [2,3].

This process is fraught with psychological challenges for patients [4]. It is often long and emotionally draining, exacerbating anxiety and stress levels [5–7]. Anxiety is particularly present at the time of embryo transfer [8,9], as this marks the end of the process and patients are concerns about possible discomfort during the procedure [10]. These factors have a direct impact on pregnancy rates [11–14]. Effective management of this anxiety is therefore essential to maximizing the chances of in vitro fertilization (IVF) success.

Based on the “blunting” theory, which suggests the use of distractions to cope with anxiety-provoking situations, various non-medicated relaxation techniques have been developed [15–17], as the device of virtual reality hypnosis (VRH). By associating a sensory immersion with hypnotic suggestions, this device distracts patients from anxiety and pain [18,19]. Early studies have already demonstrated its potential to reduce anxiety in a variety of medical contexts, including painful and stressful procedures [19–21]. In addition safety of VRH is reassuring, with studies showing that adverse effects are rare and mild, mainly limited to motion sickness, with no serious events reported [22,23].

Despite these convincing data in terms of anxiolytic efficacy and safety, the VRH device is not yet offered to women during embryo transfer in ART. This may be due to organizational constraints, limited availability, or the lack of a clearly defined framework for use, as its application in this field remains unexplored**.**

The aim of this study was to assess the feasibility of using a virtual reality medical hypnosis device for anxiolytic purposes during frozen embryo transfer (FET) and to observe its effect on subsequent pregnancy rate.

## Materials and methods

### Study design

This was a prospective, single-center, non-comparative feasibility study conducted in a French public reproductive center at the ‘Femme, Mère, Enfant’ hospital (HFME).

### Subject recruitment

Eligible women were identified during consultations dedicated to the FET prescription. Women scheduled to undergo a FET protocol between December 2023 and May 2024 were invited to participate in the trial. Eligibility criteria were women aged 18–45 years, undergoing IVF in the center, with a scheduled FET, and whose partner was willing to be present. The presence of the partner (or a third party) was required on the day of the procedure for identity verification and ethical considerations. Exclusion criteria included uncontrolled epilepsy, documented psychiatric disorders, visual or auditory impairments, claustrophobia, first embryo transfer, and fresh embryo transfer (due to organizational constraints). All participants provided an informed consent and agreed to the use of their survey data.

### Trial procedure

The VRH headset was used during the FET procedure. The device consisted of a virtual reality headset and noise-reducing headphones. A tablet was also used to control the sessions. Although sessions with a programmed duration were available, the free duration mode was used for the study.

The caregiver set up the equipment and then asked the patients to select their preferred auditory scenario (male or female voice) and visual scenario (one of 6 virtual worlds) using the tablet. The auditory scenario was a pre-recorded medical hypnosis session categorized as anxiety, while the visual scenario was a 3D world whose animations adapted to head movements thanks to special lenses and motion sensors. The session was launched using the tablet, which was connected to the headsets via Bluetooth.

The device was applied five minutes before the transfer to allow sufficient immersion time in the virtual environment [24]. It remained active throughout the transfer. Once the transfer was complete, the caregiver ended the session after five minutes using a specific gradual shutdown mode.

### Outcomes and measures

Demographic variables, data related to IVF and FET were obtained from patients’ electronic medical records. Feasibility of using VRH was assessed based on three criteria completed by the caregiver performing the FET: device availability, caregiver provision, and incident-free use. Feasibility required all the criteria to be approved. Acceptability of the procedure was assessed by recording any potential adverse effects reported by the women after the procedure. Women and caregiver satisfaction was measured using self-administered Likert scales at the end of the procedure.

Secondary exploratory endpoints included the anxiolytic effects and the pregnancy rates.

The anxiolytic effect was evaluated using the standardized State-Trait Anxiety Inventory (STAI) self-report scale, which measures anxiety in response to a particular situation [25]. This was administered to women both before and after the procedure. Consisted of 20 questions, it used a four-point Likert-type scale, with 10 items being reverse-scored. The final score could range from 0 to 80, with a higher score indicating greater anxiety.

The rate of pregnancies were determined based on the human chorionic gonadotropin (HCG) blood test, which is systematically performed ten days after the FET. A pregnancy was considered to have occurred when the HCG level was greater than 100 IU/L. An HCG level below this threshold was classified as a biochemical pregnancy. Those confirmed by ultrasound were subsequently categorized as clinical pregnancy, following the definitions of the international glossary in fertility [26].

### Statistical analysis

As the primary endpoint was device feasibility rather than clinical efficacy, and no prior data were available to estimate expected parameters, no formal sample size calculation was performed. A pragmatic sample size of 50 women over a six-month period was considered appropriate to evaluate the feasibility of the intervention, its acceptability, and to explore anxiolytic effects.

Qualitative variables were described using numbers and percentages for each category. Quantitative variables were described using the mean and standard deviation or the median and interquartile range, as appropriate. A two-sided Wilcoxon signed-rank test with a significance level of 5% was used to determine whether there was a significant difference in mean anxiety scores before and after the procedure. Effect sizes were calculated using r, derived from the test.

To analyze pregnancy rates, a comparison was made with a concurrent non-exposed cohort from the same department and inclusion period. Causal inference method was used. Each woman of the prospective interventional cohort (FET with VRH headset) was matched with a women of a concurrent non-exposed cohort (FET without VRH headset) using a propensity score (PS) constructed with logistic regression. The variables included in the PS were: female age and body mass index, AMH level, type of ART, total quantity of gonadotropin, treatment duration, sperm type, number of oocytes retrieved, number of embryos obtained and frozen, FET rank, number of embryos transferred, and stage of embryo transfer. Data processing was conducted using R software (v 4.4.0) and the ‘Matchit’ package was used for PS matching [27]. This matching was performed using the nearest neighbor method, with a 1:1 ratio, and a caliper equal to zero. After matching, a marginal comparison of the odds ratio for pregnancy with VRH was performed using the ‘Marginaleffects’ package. Only variables with no missing data were used for causal inference. A p-value < 0.05 was considered statistically significant.

### Regulation and ethical consideration

This clinical investigation falls under category 4.2 of the medical device regulations, according to the European Regulation 2017/745. The Clinical Research and Innovation Department of HCL (Hospices Civils de Lyon) provided support for this research. The study received validation from the French National Agency for the Safety of Medicines and Health Products (ANSM) on 4th of August 2023 and approved by the Regional Committee for Personal Protection (CPP) on 4th of September 2023 (ref: 2023-A01273-42).

## Results

### Study population

Between December 2023 and April 2024, 50 women were enrolled. The mean age was 33.0 years (±3.6), ranging from 27 to 40 years. The majority of them (n = 40, 80%) had experienced at least one previous pregnancy (with or without ART), including 25 women who had given birth to at least one child (Table 1). The mean duration of infertility was 69.6 months (± 3.0). A total of 78% had primary infertility, 6% had secondary infertility, and 16% had primary-secondary infertility (i.e., conception without live birth) (S1 Fig). Approximately one-third of the causes of infertility were female, one-third were male, and one-third were mixed, with the majority being endometriosis among women and oligoasthenoteratozoospermia (OAT) among men (S1 Fig). For infertility markers, the median antral follicle count was 21.0, and the median AMH was 2.8 ng/ml.

**Table 1:** Characteristics of women.

Characteristics		Women (n = 50)
Age (y), mean ± std	33.0 ± 3.6	
AMH level (ng/ml), median [IQR]	2.8 [2.0-5.6]	
Antral follicle count, median [IQR]	21.0 [14.0-30.8]	
Prior pregnancies, n (%)		
None	10 (20.0)	
1	19 (38.0)	
2	13 (26.0)	
≥ 3	8 (16.0)	
Prior live birth, n (%)		
None	25 (50.0)	
1	18 (36.0)	
2	6 (12.0)	
≥ 3	1 (2.0)	
Duration of infertility (mo), mean ± std	69.6 ± 3.0	
Cause of female infertility, N		
Endometriosis	11	
PCOS	8	
Tubal factor	8	
POI	2	
DOR	2	
Unexplained	1	
Others	6	
Cause of masculine infertility, N		
OAT	15	
Isolated teratospermia	9	
Non-obstructive azoospermia	4	
Obstructive azoospermia	1	
Others	9	

AMH: Anti-Müllerian Hormone; PCOS: Polycystic Ovary Syndrome; POI: Primary Ovarian Insufficiency; DOR: Diminished Ovarian Reserve; OAT: Oligoasthenoteratozoospermia

### ART and FET covered by the study

In this study, 80% of FET were derived from intracytoplasmic sperm injection (ICSI), with the majority using fresh spousal sperm (74%). The antagonist protocol was the most commonly used (58%), either triggered by a gonadotropin-releasing hormone (GnRH) agonist or by a double trigger (GnRH agonist and recombinant HCG). Half of the women had at least 14 oocytes at ovarian puncture and obtained at least 3 blastocysts after fertilization (Table 2). For 22% of women this was their first embryo transfer within the current IVF cycle. Endometrial preparation for FET was performed in a programmed cycle in 90% women, with a majority of transdermal estradiol patches used (n = 35). One embryo was transferred in 40 FET, while two embryos were transferred in 10 FET. Majority of the embryos were ‘day-5’ embryos, and over 80% were graded as either AA or AB (Table 2).

**Table 2:** Characteristics of the IVF and FET procedure.

Characteristics		Women (n = 50)
IVF procedure		
Type of ART, n (%)		
ICSI		40 (80.0)
c-IVF		5 (10.0)
IMSI		3 (6.0)
Oocyte recipient		2 (4.0)
Type of sperm samples, n (%)		
Fresh sperm		37 (74.0)
Fresh testicular biopsy		1 (2.0)
Frozen sperm		4 (8.0)
Frozen-thawed testicular biopsy		5 (10.0)
Sperm donors		3 (6.0)
Attempt rank, n (%)		
1		34 (68.0)
2		11 (22.0)
≥ 3		5 (10.0)
Women with prior fresh embryo transfer on this attempt, n (%)		33 (66.0)
Without pregnancies		19
With conception without live birth		8
With live birth		6
Women with prior FET on this attempt, n (%)		28 (56.0)
Without pregnancies		12
With conception without live birth		9
With live birth		7
Stimulation protocol, n (%)		
Agonist		18 (36.0)
Antagonist		29 (58.0)
r-HCG trigger		4
GnRH agonist trigger		14
Dual trigger		11
Others		3 (6.0)
Number of retrieved oocytes, median [IQR]		14.0 [9.0-20.0]
Number of mature oocytes, median [IQR]		11.5 [7.3-17.0]
Number of blastocysts, median [IQR]		5.0 [3.0-6.8]
**FET procedure**		
Transfer rank on the IVF, n (%)		
1		11 (22.0)
2		27 (54.0)
≥ 3		12 (24.0)
Endometrial preparation protocol, n (%)		
Stimulated cycle		5 (10.0)
Programmed cycle		45 (90.0)
Transdermal estradiol patch		35
Oral estradiol oral		10
Number of embryos transferred, n (%)		
SET		40 (80.0)
DET		10 (20.0)
Embryos stage, n (%)		
Day 5		49 (81.7)
Day 6		11 (18.3)
Embryos grade, n (%)		
AA	25 (42.4)	
AB	24 (40.7)	
BA	10 (16.9)	
NK	1	

ART: Assisted Reproductive Treatment; c-IVF: Conventional In Vitro Fertilization; DET: Double Embryo Transfer; GnRH: Gonadotropin-Releasing Hormone; ICSI: Intracytoplasmic Sperm Injection; IMSI: Intracytoplasmic Morphologically selected Sperm Injection; FET: Frozen Embryo Transfer; NK: Not Known; r-HCG: recombinant Human Chorionic Gonadotropin; SET: Single Embryo Transfer

### Feasibility and acceptability of the procedure

The procedure was deemed feasible with a 96% success rate and 100% acceptability (i.e., no adverse effects reported). Two transfers could not be performed optimally due to a disconnection between the headset and the driving tablet; however, this did not interrupt the ongoing scenario or disrupt the women’s immersion. On average, the device was used for 12.3 minutes (±1.3) (Table 3).

**Table 3:** Outcomes related to the intervention.

Characteristics	Women (n = 50)
Duration of the intervention (min), mean ± std	12.3 ± 1.3
Number of transfers with difficulties during the gesture*, n (%)	2 (4.0)
Feasibility of using the device, n (%)	
Availability of the device	50 (100.0)
Provision by the caregiver	50 (100.0)
Incident-free use	48 (96.0)
Acceptability of the procedure, n (%)	50 (100.0)
Caregivers’ agreement for routine use, n (%)	50 (100.0)
Number of pregnancies obtained, n (%)	25 (50.0)
Biochemical pregnancy	5
Pregnancy	20
Confirmed by ultrasound	19

* Several attempts were required

### Women and caregiver satisfaction

Women satisfaction was reported at 98%, with 39 women being very satisfied, 10 satisfied, and only one expressing no opinion (S2 Fig). VRH was considered beneficial by 42 women during the transfer procedure, 20 of whom also considered it necessary before and after the transfer (S2 Fig). Most women (n = 42, 84%) reported enjoying both the audio and visual experiences (S2 Fig). Women freewrites are reported in S1 Table. Satisfaction among caregivers reached 100%, and they had favorable opinions about using the device in routine practice.

### Anxiolytic effect

**Fig 1:**
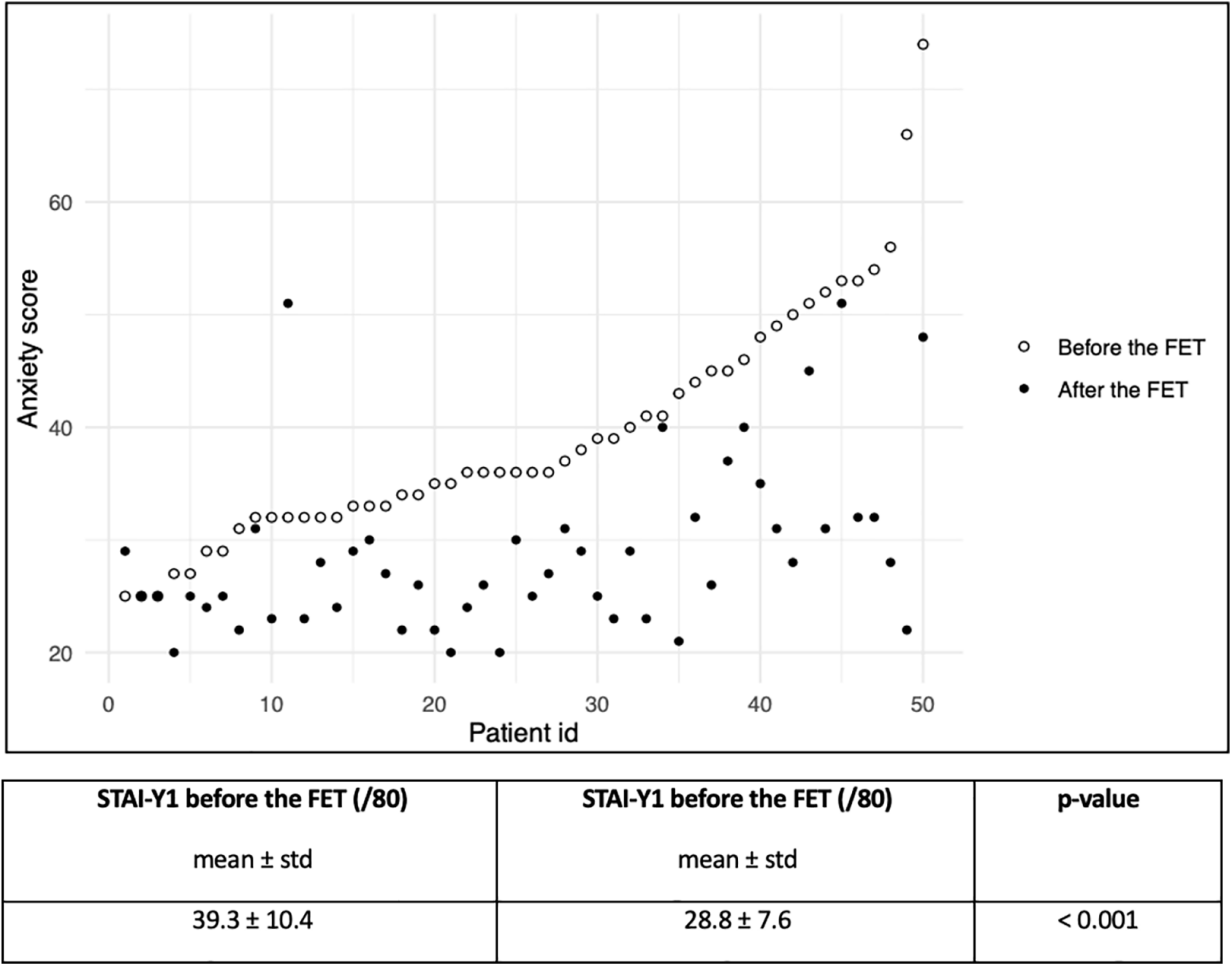
Description of anxiety scores measured before and after FET (Frozen Embryo Transfer).

The mean anxiety score after the procedure was 28.8 out of 80, compared to 39.3 out of 80 before the device was fitted, as measured by the STAI-Y1 self-report questionnaire. A statistically significant difference was observed between these two measures (p < 0.001) (Fig 1), with a large effect size (r = 0.799). Only two women reported a higher anxiety score after the procedure. One woman's score increased from 32 to 51; she found the initial relaxation exercise helpful, but experienced discomfort during speculum insertion. The other woman's score increased slightly from 25 to 29, despite feeling generally more relaxed, particularly during the uterine transfer.

### Pregnancy rates

Regarding pregnancy outcomes, considered as exploratory endpoints, 50% of women had a positive HCG test result ten days after the FET, with 20 women (40%) had HCG levels greater than 100 IU/L (Table 3). Among them, 19 were subsequently confirmed by ultrasound. During the study period, 321 women underwent FET without the VRH headset within the department. The pregnancy rate in this group was 37.1%, which was lower than that observed in our study. Of these 321 women, only 49 could be matched with women of the concurrent non-exposed cohort due to missing data from one participant who underwent a FET with an embryo imported from another center. The results of the data used for the propensity score before and after matching are shown in S2 Table. After matching and conducting a marginal comparison of the odds ratios for pregnancy rates, a positive trend was observed with a pregnancy rate of 40.8% in the VRH headset group compared to 38.8% in the concurrent non-exposed cohort. This difference was not statistically significant (OR 1.18, 95% CI 0.65–2.14; p = 0.583).

## Discussion

### Principal findings

This pilot study demonstrated the feasibility and acceptability of using VRH in routine during frozen embryo transfer. Women satisfaction reached 98%, while caregiver satisfaction was 100%, both expressing favorable opinions regarding the device's use in daily clinical practice. A significant reduction in anxiety was observed, with a 10.5-point decrease out of 80 on the STAI-Y1 score (p < 0.001). Lastly, a positive trend in pregnancy rates was noted, although it was not statistical significance.

Participants were recruited prospectively and appear to be representative of women undergoing ART. Their mean age matched that in previous studies [28], and the causes of infertility were typical, with 30% due to male factors [29,30]. Most IVF procedures used ICSI, reflecting its growing preference over conventional IVF [31,32].

To ensure comfort, only women with prior transfer experience were included, even though we finally obtained reassuring feedback on gynaecological examinations under VRH (S1 Table). It was deemed crucial for women to undergo this procedure at least once without any additional intervention, as this stage was considered a pivotal experience in their journey. Therefore, the headset was considered particularly relevant from the second trial onwards, particularly for those who had experienced multiple failures.

The feasibility of using a VRH headset during FET was high in this study. It is important to note that some physicians were already using the device, particularly during oocyte retrieval. Additionally, FET were scheduled for the late morning to avoid disrupting laboratory activities. These optimizations may have contributed to the high feasibility rate.

No adverse effects have been reported during the 50 uses. While earlier studies noted occasional fatigue or eye pain [33], recent reviews [22] and meta-analyses found no serious events [23].

Both caregivers and patients reported high levels of satisfaction with the use of headset during FET. Notably, only two procedures were considered ‘difficult,’ involving resistance during catheter insertion and requiring multiple attempts [34,35]. Furthermore, there were no cases of rigid catheter use or embryo retention. These conditions are often cited in the literature as detrimental to pregnancy rates [36–38] and are known to increase stress for both clinicians and patients [39].

The study suggested a significant reduction in anxiety levels, highlighting the anxiolytic efficacy of the device. This is particularly relevant given the psychological vulnerability of women undergoing ART [40]. Their average STAI-Y1 scores typically range from 33 to 50, with oocyte retrieval and embryo transfer being particularly stressful stages. Our baseline score of 39.3 supports this, exceeding the normative average of 35.2 in the general female population [41]. This baseline score is lower than those reported in previous studies: 41.1 by *Qu et al.* and 44.7 by *Awtani et al.* [9]. Nevertheless, the observed anxiolytic effect remains significant, underlining the robustness of the result and suggesting that it may be even more pronounced in women with higher anxiety levels. However, it is important to recall that this finding should be treated as exploratory and that the lack of a control group limits the causal interpretation.

It's also important to note that each woman acted as her own control whereas anxiety levels could decrease spontaneously after the procedure. It was observed in a study evaluating acupuncture during FET, where the control group experienced a reduction in STAI-Y1 anxiety scores despite no intervention (from 38.82 pre-FET to 31.69 post-FET) [42]. However, another study suggested that anxiety remains elevated after the FET because of the critical waiting period for pregnancy results [8].

When considering the conception rate per FET (defined as a positive HCG result), there are no French data allowing for direct comparison. A Swedish study reported a rate of 25.2% [43,44], which is lower than the 50% observed in our study. When considering the pregnancy rate per FET (defined by HCG > 100), a French multicenter retrospective study conducted between 2012 and 2016 documented a rate of 26.84% when focusing solely on artificial and stimulated cycles (43). This remains below the 40% reported in our study. When considering the clinical pregnancy rate per FET (defined as an ultrasound-confirmed), the 2014 ESHRE report showed a rate of 21.3% for France [45], while data from the French Agency of Biomedicine data showed rising rates from 22.9% in 2015 to 27.0% in 2022 [46,47]. However, this remains below the 38% reported in our study. These results should be interpreted with caution, as they may reflect the center’s specific performance. Indeed, the pregnancy rate of 37.1% for the 321 FET performed in the center over the same period was already higher than that reported elsewhere. It should also be noted that the study was not originally designed to evaluate pregnancy rates as its primary objective; these were considered exploratory and not powered for formal hypothesis testing.

### Strengths and limitations

This is the first study assessing the efficacy of the VRH device for anxiolytic purposes during FET. The prospectively enrolled population is representative of those typically treated in ART, without any selection based on the women’ anxiety levels, in order to prevent bias in the results. A key strength of this study is the use of the standardized STAI-Y1 scale to measure anxiety. This validated and reliable tool has been widely employed in studies involving infertility women, further reinforcing its relevance in this context [9,48,49]. Another strength is the active participation of all the caregivers in the center, which would facilitate the integration of this device into routine clinical practice. Furthermore, the study underscores the value of a non-pharmacological approach that enhances patient comfort and, unlike other techniques such as hypnosis, does not require specially trained personnel. This allows for large-scale implementation without significant organizational challenges. Additionally, this approach could be adapted for individuals with hearing impairments, as hypnotic induction in the virtual environment does not rely solely on sound [50]. Lastly, the use of this device shows no adverse effects on pregnancy rates and may even prove to be encouraging.

Several limitations should be highlighted. Although embryo transfer can be painful [51], which may affect anxiety and satisfaction levels, this study did not assess pain. While VRH has shown promise for pain relief in other settings [18,52], its effectiveness in this context remains unproven [53]. Therefore, it would have been valuable to assess this, even though interpreting these results without a control group would have been difficult. Another limitation is that, unlike previous studies on this topic [54,55], the study did not use a standardized VRH scenario for all women. This was due to the rapid advancement of headset technology, which could make scenarios obsolete and reduce the relevance and applicability of the results. It should also be noted that, by excluding fresh embryo transfers, the study's findings are limited to FET. However, given the procedural similarities and the positive effects observed, it is reasonable to assume that women undergoing fresh transfers could also benefit from VRH. Another limitation was that the partner was unable to use a VR headset, despite being present in the transfer room. This exclusion could potentially make the partner feel isolated, although previous studies justify this decision. Indeed, some studies have shown that there is a significant difference in the prevalence of psychological distress between partners, with women being more affected [56,57]. However, assessing partner satisfaction would have provided a more comprehensive understanding of the couple’s overall experience. Lastly, as this was a pilot study without a prospective control group, the anxiety and pregnancy outcomes remain exploratory and intended to generate hypothesis. Nevertheless, this preliminary study was essential for evaluating the feasibility and safety of this device in the context of embryo transfer.

## Conclusion

This pilot study demonstrated the feasibility and safety of using a virtual reality hypnosis device in current practice during frozen embryo transfer. Both women and caregiver satisfaction was high, reflecting broad acceptance of the device. The anxiolytic effect was observed, with a significant reduction in the STAI-Y1 score. Therefore, this device may be a useful non-medical tool for improving the overall patient experience and personalizing care pathways in assisted reproduction.

Although a positive trend in pregnancy rates was observed, this outcome was exploratory and did not reach statistical significance. Further large-scale and controlled studies are warranted to confirm these encouraging findings and to more thoroughly assess the potential impact of the device on pregnancy outcomes.

## Supporting information

S1 Fig Characteristics of the infertility. (TIFF)

S2 Fig Parameters of patient satisfaction with device use. (TIFF)

S1 Table Freewriting commentary by the participants. (DOCX)

S2 Table Distribution of matching parameters before and after matching process. (DOCX)
