# Supplementary figures and images for: Use of virtual reality medical hypnosis for anxiolytic purposes during frozen embryo transfer: A prospective pilot study
Source: PLoS One. 2026 May 26;21(5):e0350101. doi: 10.1371/journal.pone.0350101 (PMC13210230; doi:10.1371/journal.pone.0350101)

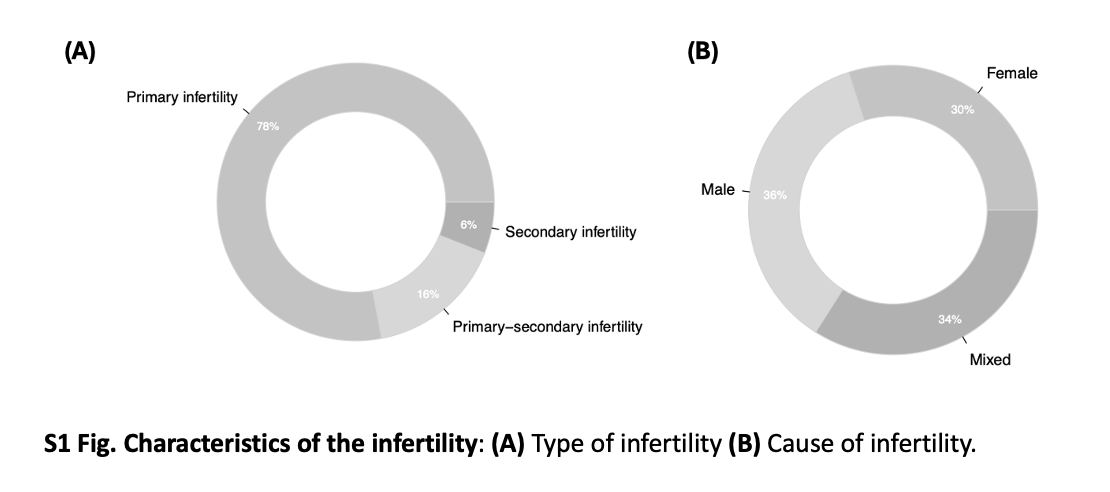

Supplement: S1 Fig — (TIFF) [file pone.0350101.s001.tiff]

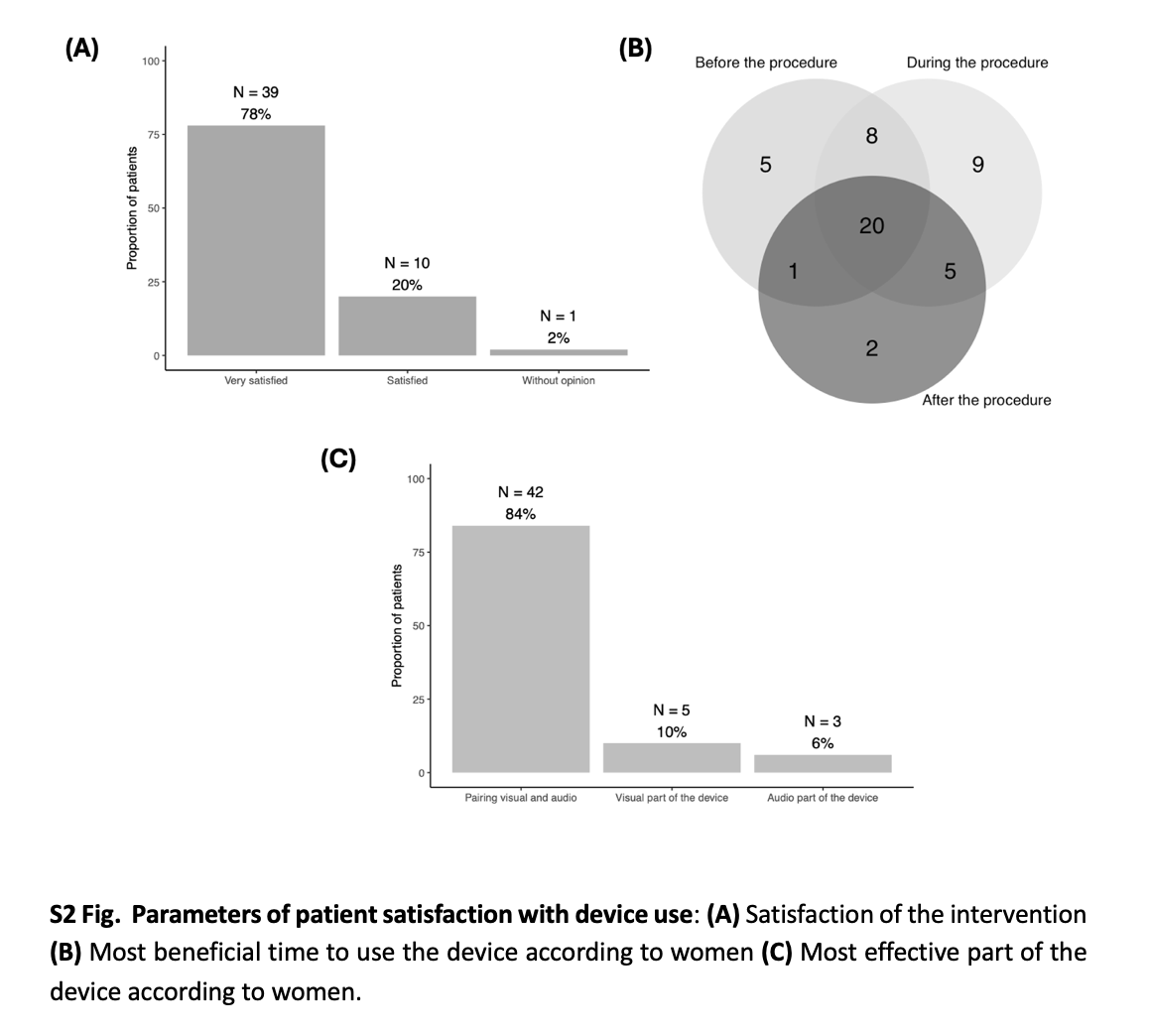

Supplement: S2 Fig — (TIFF) [file pone.0350101.s002.tiff]

**S1 Table. Freewriting commentary by the participants.**


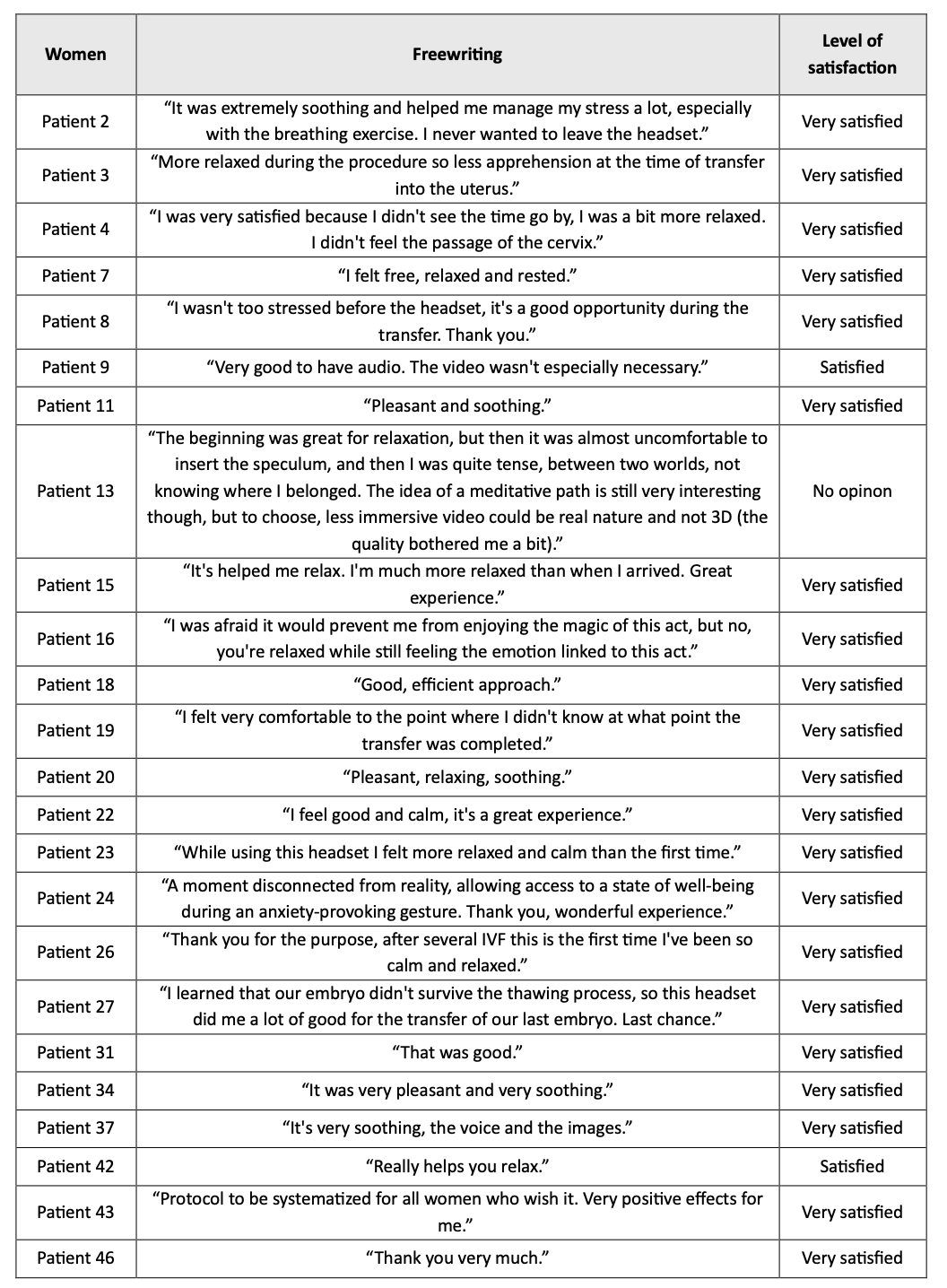

Supplement: S1 Table — (DOCX) [file pone.0350101.s003.docx]
