# Supplementary material for: Use of virtual reality medical hypnosis for anxiolytic purposes during frozen embryo transfer: A prospective pilot study
Source: PLoS One. 2026 May 26;21(5):e0350101. doi: 10.1371/journal.pone.0350101 (PMC13210230; doi:10.1371/journal.pone.0350101)
Supplement: S2 Table — (DOCX) [file pone.0350101.s004.docx]

**S2 Table. Distribution of matching parameters before and after matching process.**


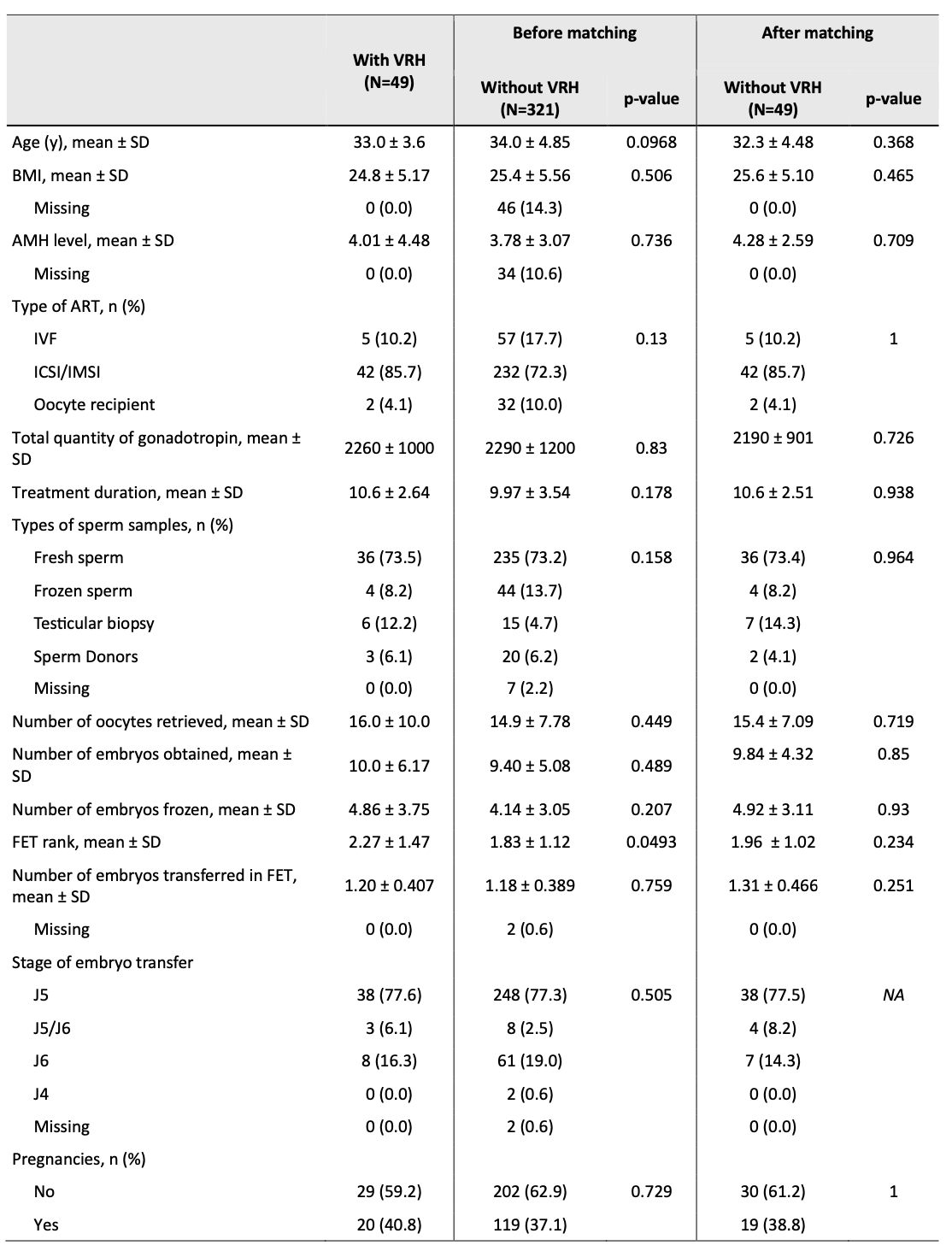


BMI: Body Mass Index; ART: Assisted Reproductive Treatment; IVF: In Vitro Fertilization; ICSI: Intracytoplasmic Sperm Injection; IMSI: Intracytoplasmic Morphologically selected Sperm Injection; FET: Frozen Embryo Transfer
